# Sex Differences in HIV Testing among Older Adults in Sub-Saharan Africa: A Systematic Review

**DOI:** 10.1155/2021/5599588

**Published:** 2021-08-21

**Authors:** Akalewold T. Gebremeskel, Nathali Gunawardena, Olumuyiwa Omonaiye, Sanni Yaya

**Affiliations:** ^1^School of International Development and Global Studies, University of Ottawa, Ottawa, Ontario, Canada; ^2^Faculty of Health Sciences, University of Ottawa, Ottawa, Ontario, Canada; ^3^Faculty of Medicine, McGill University, Montreal, Quebec, Canada; ^4^Centre for Quality and Patient Safety Research, School of Nursing and Midwifery, Deakin University, Melbourne Burwood, Victoria, Australia; ^5^Centre for Nursing and Midwifery Research, James Cook University, Townsville, Queensland, Australia; ^6^University of Parakou, Faculty of Medicine, Parakou, Benin

## Abstract

**Background:**

Despite being sexually active and engaging in risky sexual behaviours similar to young adults, older adults (50 years or older) are less likely to receive HIV testing, and disaggregated data are still scarce about HIV prevention and treatment in this vulnerable population in sub-Saharan Africa (SSA). This systematic review is aimed at examining sex differences in HIV testing and counseling (HTC) among older adults in SSA.

**Methods:**

A systematic search of four databases, namely, MEDLINE (Ovid), EMBASE (Ovid), Web of Science, and Global Health, was conducted from 2000 to January 2020. The primary outcome of interest for this study was gender differences in HTC among older adults in SSA. Observational studies including cross-sectional, retrospective, and prospective cohort studies were included. Eligible studies must have reported sex differences in HIV testing uptake in a standard HTC service among older adults in SSA.

**Results:**

From the database search, 4143 articles were identified. Five studies were ultimately included in the final review. Of the 1189 participants, 606 (51.1%) and 580 (48.9%) were female and male, respectively. The review findings suggested that both men and women preferred HTC providers that are the same sex as them with women additionally preferring a provider who is also of a similar age. Men and women differed in their pathways to getting tested for HIV. The review documented mixed results with regard to the associations between sex of older adults and uptake of HTC. Older adult HTC uptake data are limited in scope and coverage in sub-Saharan Africa.

**Conclusion:**

This review revealed shortage of evidence to evaluate optimum HTC utilization among older adults. Few studies examined sex differences in HIV testing among older adults in the region. There is a need for stakeholders working in the area of HIV prevention and treatment to focus on older adult health utilization evidence organization, disaggregated by age and sex. Hence, high-quality research designs are needed on the topic in order to generate good quality evidence for targeted interventions to improve HTC among older adults in sub-Saharan Africa.

## 1. Background

Human Immunodeficiency Syndrome (HIV) remains a leading cause of morbidity and mortality in sub-Saharan Africa (SSA) [1, 2]. Globally, according to the United Nations Programme on HIV and AIDS (*UNAIDS*) 2021 epidemiological report, 37.6 million people are living with HIV in 2020, over two-thirds of whom are in SSA [1]. In 2017, it was estimated that four million people aged 50 years and older were living with HIV in SSA [3]. In this region, the main target for HIV/AIDS preventive care is younger people, and older adults' information is limited. Also, most population-based HIV prevalence surveys in SSA have not included older adults, limiting the accuracy of estimates in this age group [4].

According to the United Nations Department of Economic and Social Affairs, Population Division, SSA is projected to be home to 161 million older persons by 2050, which is faster than that experienced by any other regions [5]. In order to respond and address this continued growing of ageing and vulnerable older people in SSA and in the context of meeting the Sustainable Development Goals (SDGs) 2030, goal 3 [6], which is to ensure healthy lives and promote well-being for all at all ages, it is crucial that a systematically organized evidence-based approach is undertaken in order to provide useful information about the health and well-being of older adults in SSA in relation to HIV preventive care and support. HIV testing and counseling (HTC) play a critical role in HIV prevention, care, and treatment as it helps identify those who have HIV and provide the platform for linkage to information, services, and support that is required after a positive or negative test result [7].

As evidence suggests, similar to younger adults, older adults (50 and older) continue to be vulnerable—sexually active, participate in casual sex, engage inconsistent condom use, and sex with more than one partner [8–13]. Despite these risk factors, older adults are less likely to be tested for HIV compared to their younger counterparts [8, 14–16]. Despite the availability of studies examining sex differences in HIV testing within the SSA region, they do not examine sex differences in testing among older adults. However, there are currently few studies that have examined HIV infection in older adults, and the few that do exist have been conducted in high-income countries [14, 16]. Most studies consider older people as a single category, typically including all adults aged 50 and over [2].

The lack of sex and age disaggregated information among older adults is problematic in SSA [2, 17–21], given that this type of data is essential to better understand HIV service utilization and preferences that can help develop appropriate evidence-based responses and policies to ensure health access equality for older adults in this region. The aim of this systematic review is to identify and collect all available data both from qualitative and quantitative studies that examined sex differences in HIV testing among older adults in SSA. It is expected that a better understanding of HTC among older adults would aid policy makers and practitioners to promote older people age and sex aligned HTC service and data access strategies in the region.

## 2. Methods

### 2.1. Study Design

The protocol for this systematic review was registered within the PROSPERO database (CRD42020172737). The protocol was designed and written according to the Preferred Reporting Items for Systematic Review and Meta-Analysis Protocols (PRISMA-P) guideline for reporting systematic reviews [22] (see checklist in Additional file 1). This review was conducted as per the Cochrane Collaboration Handbook of Systematic Reviews [23], and the findings were reported in accordance with the reporting guidance provided in the PRISMA statement [24].

### 2.2. Inclusion Criteria Type of Study

The study includes observational studies (cross-sectional, retrospective, and prospective cohort studies). Eligible studies must have reported sex differences in HIV testing uptake in a standard of HTC service among older adults in SSA.

### 2.3. Type of Participants

Studies focusing on male and female older adults who received HTC in SSA will be considered.

### 2.4. Type of Intervention(s)

There was no specific intervention targeted for this study; however, we considered the usual standard of HTC services at stand-alone, mobile, home to home, and health facility-based.

### 2.5. Type of Outcome

The primary outcomes of interest for this study were sex differences in HTC among older adults in SSA.

### 2.6. Exclusion Criteria

Studies that did not report sex differences in HTC among older adults in SSA were excluded. In addition, HTC studies conducted outside of SSA countries; nonprimary literature, such as reviews, dissertations, theses, editorials, protocol studies, and clinical guidelines; non-peer-reviewed journal articles; grey literature; PhD theses; and conference proceedings were also excluded.

### 2.7. Search Methods for Identification of Studies

#### 2.7.1. Electronic Searches

The primary source of literature was a structured search of major electronic databases : MEDLINE (Ovid), EMBASE (Ovid), Web of Science, and Global Health for studies reporting HIV data from 2000 to 2020. We searched for the following terms (and their related synonyms): sub-Saharan Africa, older adults, HIV/AIDS, testing, and gender differences. The searches were designed and conducted by the review team which included two experienced public health researchers in collaboration with a health science librarian. We performed handsearching of the reference lists of included studies (see search strategy in Additional file 2).

#### 2.7.2. Selection of Studies

**Figure 1:**
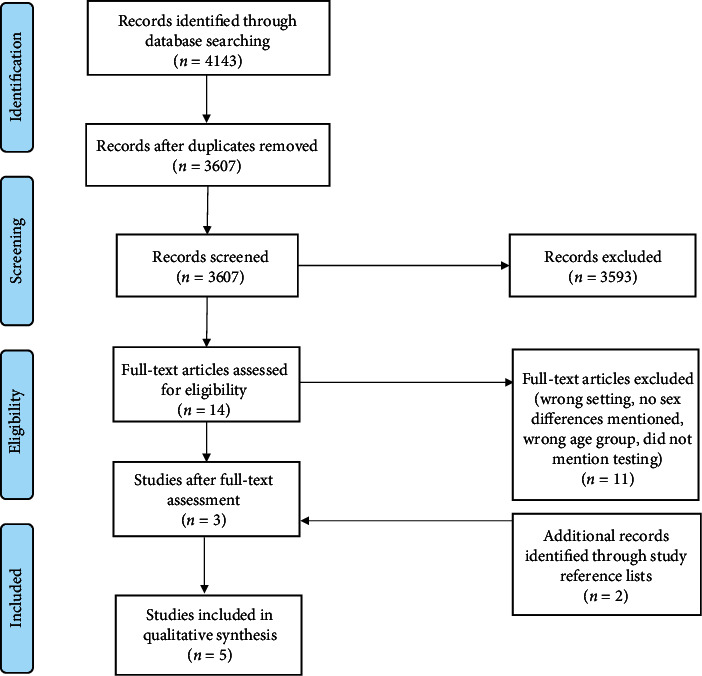
PRISMA flow diagram of systematic search results and study selection.

All the identified studies of different sources were imported into the Covidence online citation management software, and duplicates were removed. Two reviewers (AG and NG) independently screened the titles and abstracts according to a predefined inclusion criterion checklist and exclude unrelated ones. Disagreements were resolved through discussion with SY and OO. The PRISMA (Preferred Reporting Items for Systematic Review and Meta-Analyses) flowchart [23] was used to document the selection process (see Figure 1).

#### 2.7.3. Data Extraction

The authors adopted a data collection form based on the needs of the review from a standardized data extraction form from the Cochrane Library. The form was designed and used to extract information from each study. The content of each of the included studies was extracted by two authors, independently, and potential conflicts were resolved through discussion with SY and OO (see Table 1).

#### 2.7.4. Appraisal of Study Quality

Methodological rigor in this review was conducted by having two independent reviewers critically appraise the methodological validity of the included studies. The authors assessed the reporting of included qualitative studies using the criteria based on the Critical Appraisal Skills Programme (CASP) 10 Questions for Qualitative Research [25] (see Table 2 in Additional file 3). CASP was selected due to its extensive use in previous systematic reviews of qualitative studies. The domains of the CASP checklist helped assess the credibility of the qualitative findings and the rigor of the studies. The 10 questions were designed as prompts to guide reviewers in critically reading the reports, and each question was given a score of “yes” or “no.” Examples of the 10 questions asked in the CASP checklist include “Was there a clear statement of the aims of the research? Is a qualitative methodology appropriate? Was the research design appropriate to address the aims of the research? Was the recruitment strategy appropriate to the aims of the research? Was the data collected in a way that addressed the research issue? Has the relationship between researcher and participants been adequately considered? Have ethical issues been taken into consideration? Was the data analysis sufficiently rigorous? Is there a clear statement of findings? How valuable is the research?”

The authors assessed the reporting of included quantitative studies using the criteria based in the Quality Assessment Tool for Quantitative Studies by the Effective Public Health Practice Project [26] (see Table 3 in Additional file 3). Each study was assessed based on six domains and given a score of “strong,” “moderate,” or “weak.” Then, an overall score was given for each study based on the sum of values given on the six domains. Global rating for the studies was determined by “strong” (no weak ratings), “moderate” (one weak rating), and “weak” (two or more weak ratings). Studies were not excluded or weighted based on the quality of the reporting assessments. The results of the appraisals and assessments were instead used to inform data interpretation and ultimately determine trustworthiness of review findings and conclusions. Differences in the quality assessment were resolved by discussion among all of the authors.

#### 2.7.5. Data Synthesis

We synthesized data using textual narrative summaries [27]. Studies were summarized and presented into two categories. Findings from studies that reported quantitative differences in HIV testing between men and women age 50 or older were summarized together while findings from studies that reported qualitative differences were summarized together.

## 3. Results

### 3.1. Search Results

The literature search returned 4143 studies, with 3607 remaining after duplicate removal; 3593 studies were excluded after abstract screening, and thus, 14 full articles were screened. Following 11 exclusions at the full-text level, and inclusion of 2 additional studies through review of reference lists, 5 studies were included in the final review. Figure 1 shows the PRISMA flow diagram depicting the study screening and inclusion process.

### 3.2. Study Characteristics

All studies were cross-sectional by design, two studies used a qualitative approach [28, 29], two studies used a quantitative approach [30, 31], and one study used a mixed methods approach [32]. Three studies were conducted in Eastern Africa (Kenya, Tanzania, and Uganda). Two studies were conducted in Southern Africa (Botswana and South Africa) (Table 1).

### 3.3. Participant Characteristics

Participants in all 5 studies were women and men aged ≥50 years. In two studies [28, 29], all participants were HIV positive. In one study [29], the participants were participating in HIV care at clinics. Sex difference in HTC service uptake among seniors was examined; of the total of 1189 older adultparticipants, 606 were female (51.1%) and 580 were male (48.9%).

### 3.4. Qualitative Differences in HIV Testing

Two studies reported qualitative differences in HIV testing between male and female older adults (age ≥ 50) [28, 29]. In-depth interviews and focus groups conducted in Kenya revealed that in terms of HTC, male participants unanimously expressed the need to have a male provider who can discuss male-related sex issues which were considered “private,” including erection difficulties and sexually transmitted diseases [28]. In contrast, female participants expressed wanting a provider who is also of a similar age to them in addition to being the same gender [28]. Qualitative study conducted in South Africa examined differences in what led men and women to seek HIV testing [30]. In the aforementioned qualitative study, in-depth interviews and key informant interviews revealed that most women were tested for HIV while undergoing on-going care for another chronic condition and most women were tested after a provider suggested they do so [29]. In comparison, men were most often tested as a result of their wives being tested. In addition, men were also tested for HIV along with TB when they came to a clinic for coughing or other TB-related symptoms [29].

### 3.5. Quantitative Differences in HIV Testing

Three studies reported quantitative differences in HIV testing between male and female elders (age ≥ 50) [29, 31, 32]. In a cross-sectional study conducted in Tanzania, being male was associated with lower odds of HTC uptake (AOR = 0.5, 95%CI = 0.3-0.7, *p* < 0.01) [29] and female gender was associated with increased HTC uptake. While in Botswana, a cross-sectional study showed that females are as likely to use HTC services as males (OR = 1) and that females are less likely to take an HIV test than men (OR = 0.762) [30]. In addition to quantitative differences in HTC uptake, the study conducted in Botswana also found that more males (78.2%) than females (70%) found HTC programs helpful [31]. However, a cross-sectional study conducted in Uganda found that sex was not significantly associated with HIV testing in the 12 months prior to the study [32].

## 4. Discussion

This systematic review is aimed at identifying and describing qualitative and quantitative differences in HIV testing among older adults in sub-Saharan Africa. Overall, the body of research on the topic was very limited. Few studies examined sex differences in HIV testing among older adults in SSA. We were able to identify very few studies (five) that looked at HIV testing among older adults in SSA, and out of the few studies we did find that addressed sex differences in HIV testing among this population, only one study set out to answer this research question as their main focus while others focused on sex differences as a subpoint in their overall paper. The lack of studies conducted on our research question corroborates the existing findings that there is very little HIV data focused on older adults in SSA [2, 4, 8, 16] despite the region being home to the five countries with the highest number of older adults with HIV [14]. The lack of data and research may be the result of HIV responses being targeted to younger people while neglecting older adults [20, 33–35].

The SDG 17, target 17.18 acknowledges and recommends capacity building for developing countries in order to substantially increase their capability to provide high-quality data disaggregated by income, gender, age, race, ethnicity, migratory status, disability, and geographic location. In the SDG 17, target 17.18 acknowledges and recommends capacity building efforts to increase the availability of data disaggregated by income, gender, age, race, ethnicity, migratory status, disability, and geographic location in developing countries to ensure that no one is left behind [6]. Hence, the effort to address the disappointing inequality in HIV testing and related service access among older adults is dependent on the generation and provision of high-quality data to facilitate evidence-based actions that can be used at national, regional, and international levels to monitor the progress of preventive HIV care and support for older adults at both community and health facility levels. There is a need for stakeholders working in the area of HIV prevention and treatment to focus on older adult health utilization evidence organization, disaggregated by age and sex.

The quantitative findings produced mixed results in relation to sex differences in HIV testing among older adults in SSA. One study found that being female was associated with greater HIV testing; one found that being male was associated with greater HIV testing, while another found no differences between men and women in terms of HIV testing. These findings conflict with existing studies that have examined sex differences in HIV testing among younger people (less than age 50) in SSA, where there has generally been a singular consensus that women are more likely to get tested for HIV [36–39]. One of the qualitative findings of our review consistent with existing research was that both men and women prefer an HTC provider of the same gender as themselves. This is in line with other studies that have found that patients prefer providers of the same gender [40, 41]. This same-gender preference may be due to differences in communication styles between male and female patients [42–44].

This systematic review has several different limitations. The first limitation is that data sources for the study were published in four online databases including MEDLINE, Global Health, Web of Science, and EMBASE. Other relevant studies describing HIV testing differences among older people in SSA may have been missed for the reason that they may have been included in other databases. Grey literature, reports, conference abstracts, and other non-peer-reviewed studies were not included in this review, adding to the limitations. Lastly, despite using a rigorous search strategy, our review resulted in very few studies, some with conflicting findings that made it difficult to draw concrete conclusions and make recommendations. Nevertheless, our systematic review has identified a large gap in available research on sex differences in HIV testing in SSA among older adults that could encourage more primary studies to be conducted on the topic.

## 5. Conclusion

The review revealed shortage of evidence to evaluate optimum HTC utilization among older adults. There is few available evidence that exists examining sex differences in HIV testing among older adults in SSA. The limited studies that we have examined showed mixed results. The national level public health surveys, health organization administrative data, and academic and related research stakeholders need to focus on older adult's health utilization evidence, disaggregated by age and sex to improve data access in scope and coverage. More primary research is also needed on the topic in order to create targeted interventions that could help improving HTC in older adults in SSA and in the context of SDGs, leave no one behind.

## Figures and Tables

**Table 1:** Study information.

Authors	Kiplagat and Huschke, 2018 [28]	Muiruri et al., 2019 [29]	Schatz and Knight, 2018 [30]	Ama et al., 2015 [31]	Wandera et al., 2020 [32]
Title	HIV testing and counselling experiences: a qualitative study of older adults living with HIV in western Kenya	Individual and partner characteristics associated with HIV testing and counseling uptake among individuals 50 years or older in Tanzania	“I was referred from the other side”: gender and HIV testing among older South Africans living with HIV	Knowledge and utilization of voluntary counselling and testing services for HIV by older adults (50 years and over) in Botswana	Prevalence and determinants of recent HIV testing among older persons in rural Uganda: a cross-sectional study
Database search or reference list	Database	Database	Database	Reference list	Reference list
Country	Kenya	Tanzania	South Africa	Botswana	Uganda
Urban/rural?	Both	Rural	Urban	Both	Rural
Number of participants	57	600	Not stated	609	649
Number of female participants	27	289	10	371	334
Number of male participants	30	311	Exact number not stated. However, it is mentioned that the number is similar to the number of women	238	315
Methodology	Qualitative	Quantitative	Qualitative	Quantitative	Mixed methods (but chose only to report the quantitative findings)
Study design	Cross-sectional	Cross-sectional	Cross-sectional	Cross-sectional	Cross-sectional
Participant characteristics	HIV-infected men and women aged ≥50 years at the time of HIV care enrollment and receiving care, currently in care at two participating outpatient HIV clinics (one urban and one rural), had been followed up for at least 1 year	Older adults ≥ 50 years of age	Women and men aged 50 and over who are living with HIV from two urban townships outside of Cape Town	Older people (≥50 years) living in four purposively sampled districts in Botswana	Older men and women age 50 years and older, from central (Masaka district) and western (Hoima district) Uganda
Recruitment	Participants who were currently in care were selected from one urban and one rural facility	Analysis of data from a prior cross-sectional survey followed by multistage sampling where individuals aged 50 years and older were randomly selected from village registers and then visited at their home and invited to participate	Participants were recruited through nonprobability sampling methods, making connections through a survey list from a local NCD research project, as well as convenience sampling and referrals by health workers at HIV clinics	Respondent-driven sampling (RDS)	Multistage stratified cluster sampling design
Data collection methods	In-depth interviews, focus group discussions	Surveys, HIV testing to determine status	In-depth interviews, key informant interviews	Questionnaire administered through in-person interviews	Focus group discussions, in-depth interviews, survey results
Data analysis methods	Thematic content analysis	Logistic regression	Grounded theory to specify emerging themes	Binary logistic regression	Frequency distributions, chi-squared tests, and multivariable logistic regression
Sex differences in testing	Male participants unanimously expressed the need to have a male provider who can discuss male-related sexual issues which were considered “private,” including erection difficulties and sexually transmitted diseases. In addition to wanting a provider of the same gender, female participants also expressed wanting a provider of a similar age to them	Being male was associated with lower odds of HTC uptake (AOR = 0.5, 95% CI 0.3-0.7). Female gender was associated with increased HTC uptake	Nearly all of the women were tested for HIV while undergoing ongoing care for another chronic condition, and most women were tested after a provider suggested they do so. In comparison, men were most often tested as a result of their wives being tested first which prompted them to get tested. In addition, men also were tested for HIV along with TB when they came to a clinic for coughing or other TB-related symptoms	Of those who had participated in the VCT programme (55 males and 60 females), more males (78.2%) than females (70%) found the programme very helpful; females are less likely to take an HIV test than males (OR = 0.762); females are as likely to use the VCT services as the males (OR = 1.0)	Sex was not significantly associated with HIV testing in the 12 months prior to the study

## Data Availability

Data sharing is not applicable to this article as no datasets were generated or analyzed during the current study.

## Abbreviations

AIDS: Acquired Immunodeficiency Syndrome
HIV: Human Immunodeficiency Virus
HTC: HIV testing and counseling
PRISMA-P: Preferred Reporting Items for Systematic Review and Meta-Analysis Protocols
PROSPERO: International Prospective Register of Systematic Reviews
SDGs: Sustainable Development Goals
SSA: Sub-Saharan Africa
UN: United Nations
WHO: World Health Organization.

## Supplementary Materials

Supplementary 1 Additional file 1: PRISMA checklist.

Supplementary 2 Additional file 2: sample search strategy (MEDLINE).

Supplementary 3 Additional file 3: summary of quality scores.

## References

[1] United Nations Programme on HIV and AIDS (UNAIDS) Global HIV/AIDS and AIDS Statistics. https://www.unaids.org/en/resources/fact-sheet.

[2] Lloyd-Sherlock P., Amoakoh-Coleman M. (2020). A critical review of intervention and policy effects on the health of older people in sub-Saharan Africa. *Social Science & Medicine*.

[3] United Nations Programme on HIV and AIDS (UNAIDS) http://aidsinfo.unaids.org/.

[4] Harris T. G., Rabkin M., El-Sadr W. M. (2018). Achieving the fourth 90: healthy aging for people living with HIV. *AIDS*.

[5] United Nations WPA2017_Report.pdf. https://www.un.org/en/development/desa/population/publications/pdf/ageing/WPA2017_Report.pdf.

[6] United Nations (2015). *A/RES/70/1 Transforming our world: the 2030 Agenda for Sustainable Development*.

[7] Fonner V. A., Denison J., Kennedy C. E., O'Reilly K., Sweat M. (2012). Voluntary counseling and testing (VCT) for changing HIV-related risk behavior in developing countries. *Cochrane Database of Systematic Reviews*.

[8] Mutevedzi P. C., Newell M.-L. (2011). A missing piece in the puzzle: HIV in mature adults in sub-Saharan Africa. *Future Virology*.

[9] Negin J., Geddes L., Brennan-Ing M., Kuteesa M., Karpiak S., Seeley J. (2016). Sexual behavior of older adults living with HIV in Uganda. *Archives of Sexual Behavior*.

[10] UN General Assembly (2015). *Transforming our world: the 2030 Agenda for Sustainable Development*.

[11] Freeman E. (2016). *Attitudes towards sexual health and sex behaviour among older adults with HIV in rural southern Malawi*.

[12] Pilowsky D., Wu L.-T. T. (2015). Sexual risk behaviors and HIV risk among Americans aged 50 years or older: a review. *Substance Abuse and Rehabilitation.*.

[13] Rosenberg M., Gómez-Olivé F., Rohr J. (2016). Sexual behaviors and HIV status: a population-based study among older adults in rural South Africa. *Journal of Acquired Immune Deficiency Syndromes*.

[14] Negin J., Cumming R. G. (2010). HIV infection in older adults in sub-Saharan Africa: extrapolating prevalence from existing data. *Bulletin of the World Health Organization*.

[15] Gebo K. A. (2008). Epidemiology of HIV and response to antiretroviral therapy in the middle aged and elderly. *Aging Health*.

[16] Gómez-Olivé F. X., Houle B., Rosenberg M. (2020). Brief report: HIV incidence among older adults in a rural South African setting: 2010-2015. *Journal of Acquired Immune Deficiency Syndromes*.

[17] Bhavan K. P., Kampalath V. N., Overton E. T. (2008). The aging of the HIV epidemic. *Current HIV/AIDS Reports*.

[18] Navarro G., Nogueras M. M., Segura F. (2008). HIV-1 infected patients older than 50 years. PISCIS cohort study. *The Journal of Infection*.

[19] Cahill S., Valadéz R. (2013). Growing older with HIV/AIDS: new public health challenges. *American Journal of Public Health*.

[20] Schmid G. P., Williams B. G., Garcia-Calleja J. M. (2009). The unexplored story of HIV and ageing. *Bulletin of the World Health Organization*.

[21] Knight L., Mukumbang F. C., Schatz E. (2018). Behavioral and cognitive interventions to improve treatment adherence and access to HIV care among older adults in sub-Saharan Africa: an updated systematic review. *Systematic Reviews*.

[22] Moher D., Shamseer L., Clarke M. (2015). Preferred reporting items for systematic review and meta-analysis protocols (PRISMA-P) 2015 statement. *Systematic Reviews*.

[23] Moher D., Liberati A., Tetzlaff J., Altman D. G., The PRISMA Group (2009). Preferred Reporting Items for Systematic Reviews and Meta-Analyses: the PRISMA statement. *PLOS Medicine*.

[24] Higgins J. P. T., Altman D. G., Gotzsche P. C. (2011). The Cochrane Collaboration’s tool for assessing risk of bias in randomised trials. *BMJ*.

[25] Critical Appraisal Skills Programme (2018). CASP qualitative checklist: 10 questions help you make sense of a qualitative research. *Summertown Pavilion*.

[26] Effective Public Health Practice Project Quality assessment tool for quantitative studies. https://merst.ca/wp-content/uploads/2018/02/quality-assessment-tool_2010.pdf.

[27] Popay J., Roberts H., Sowden A. (2006). *Guidance on the conduct of narrative synthesis in systematic reviews: a product of the ESRC methods programme (Version I)*.

[28] Kiplagat J., Huschke S. (2018). HIV testing and counselling experiences: a qualitative study of older adults living with HIV in western Kenya. *BMC Geriatrics*.

[29] Muiruri C., Swai S. J., Ramadhani H. O. (2019). Individual and partner characteristics associated with HIV testing and counseling uptake among individuals 50 years or older in Tanzania. *International Journal of STD & AIDS*.

[30] Schatz E., Knight L. (2018). “I was referred from the other side”: gender and HIV testing among older South Africans living with HIV. *PLoS One*.

[31] Ama N. O., Shaibu S., Ama H. A. (2015). Knowledge and utilization of voluntary counselling and testing services for HIV by older adults (50 years and over) in Botswana. *Journal of AIDS & Clinical Research*.

[32] Wandera S. O., Kwagala B., Maniragaba F. (2020). Prevalence and determinants of recent HIV testing among older persons in rural Uganda: a cross-sectional study. *BMC Public Health*.

[33] Wallrauch C., Bärnighausen T., Newell M.-L. (2010). HIV prevalence and incidence in people 50 years and older in rural South Africa. *South African Medical Journal*.

[34] Danielson M. E., Justice A. C. (2001). Veterans aging cohort study (VACS) meeting summary. *Journal of Clinical Epidemiology*.

[35] Nguyen N., Holodniy M. (2008). HIV infection in the elderly. *Clinical Interventions in Aging*.

[36] Gebregziabher M., Dai L., Vrana-Diaz C., Teklehaimanot A., Sweat M. (2018). Gender disparities in receipt of HIV testing results in six sub-Saharan African countries. *Health Equity*.

[37] Treves-Kagan S., el Ayadi A. M., Pettifor A. (2017). Gender, HIV testing and stigma: the association of HIV testing behaviors and community-level and individual-level stigma in rural South Africa differ for men and women. *AIDS and Behavior*.

[38] Ha J. H., van Lith L. M., Mallalieu E. C. (2019). Gendered relationship between HIV stigma and HIV testing among men and women in Mozambique: a cross-sectional study to inform a stigma reduction and male-targeted HIV testing intervention. *BMJ Open*.

[39] Schell E. S., Geoffroy E., Phiri M., Bvumbwe A., Weinstein J., Jere J. M. (2016). Cracking the code: getting men tested in rural Africa. *AIDS*.

[40] Schmittdiel J. A., Traylor A., Uratsu C. S., Mangione C. M., Ferrara A., Subramanian U. (2009). The association of patient-physician gender concordance with cardiovascular disease risk factor control and treatment in diabetes. *Journal of Women's Health*.

[41] Chesang K., Hornston S., Muhenje O. (2017). Healthcare provider perspectives on managing sexually transmitted infections in HIV care settings in Kenya: A qualitative thematic analysis. *PLoS Medicine*.

[42] Bertakis K. D., Azari R. (2007). Patient gender and physician practice style. *Journal of Women's Health*.

[43] Elderkin-Thompson V., Waitzkin H. (1999). Differences in clinical communication by gender. *Journal of General Internal Medicine*.

[44] Weisman C. S. (1987). Communication between women and their health care providers: research findings and unanswered questions. *Public Health Reports*.

