# Supplementary material for: Sex Differences in HIV Testing among Older Adults in Sub-Saharan Africa: A Systematic Review
Source: Biomed Res Int. 2021 Aug 21;2021:5599588. doi: 10.1155/2021/5599588 (PMC8427674; doi:10.1155/2021/5599588)
Supplement: Supplementary 2 — Additional file 2: sample search strategy (MEDLINE). [file 5599588.f2.docx]

**Additional file 2.** Sample Search Strategy (Medline)

1. (Angola or Benin or Botswana or Burkina Faso or Burundi or Cameroon or Cape Verde or Central African Republic or Chad or Congo or Ivory Coast or Djibouti or Eritrea or Ethiopia or Gabon or Gambia or Ghana or Guinea or Kenya or Lesotho or Liberia or Madagascar or Malawi or Mali or Mauritania or Mauritius or Mozambique or Namibia or Niger or Nigeria or Reunion or Rwanda or Sao Tome) and Principe) or Senegal or Seychelles or Sierra Leone or Somalia or South Africa or Sudan or Swaziland or Tanzania or Togo or Uganda or Zambia or Zimbabwe).ti,ab.
2. Exp “Africa South of the Sahara”/
3. or/1-2
4. exp Aged/
5. Middle Aged/
6. (elderl* or senior* or geriatric* or older adult*).ti,ab.
7. Middle age*.ti,ab.
8. Or/4-7
9. Exp HIV/ or exp HIV Infections/
10. HIV.ti,ab.
11. Human Immunodeficiency Virus.ti,ab.
12. (HIV adj2 (infection* or coinfection*)).ti,ab.
13. (Acquired Immune Deficiency Syndrome or AIDS).ti,ab.
14. Or/9-13
15. (Screen* or test* or counsel* or HCT).ti,ab.
16. (gender adj2 difference*).ti,ab.
17. (m?n or male*or (wom?n or female*)).ti,ab.
18. Male/ or Female/
19. 16 or 17 or 18
20. 3 and 8 and 14 and 15 and 19
