# Supplementary material for: Sex Differences in HIV Testing among Older Adults in Sub-Saharan Africa: A Systematic Review
Source: Biomed Res Int. 2021 Aug 21;2021:5599588. doi: 10.1155/2021/5599588 (PMC8427674; doi:10.1155/2021/5599588)
Supplement: Supplementary 3 — Additional file 3: summary of quality scores. [file 5599588.f3.docx]

**Additional file 3.** Summary of quality scores

Qualitative studies based on 10 CASP checklist questions

| **Qualitative studies** | Kiplagat and Huschke | Schatz and Knight |
| --- | --- | --- |
| Was there a clear statement of research aims | Y | Y |
| Is a qualitative methodology appropriate | Y | Y |
| Was the research design appropriate to address the aims of the research | Y | Y |
| Was the recruitment strategy appropriate to the aims of the research | Y | Y |
| Was the data collected in a way that addressed the research issue | Y | Y |
| Has the relationship between researcher and participants been adequately considered | N | N |
| Have ethical issues been taken into consideration | Y | Y |
| Was the data analysis sufficiently rigorous? | Y | Y |
| Is there a clear statement of findings | Y | Y |
| How valuable is the research? | Very valuable | Very valuable |

Y = Yes, N = No

Quality scores based on Effective Public Health Practice Project (EPHPP) Quality Assessment Tool for Quantitative Studies

| **Quantitative Studies** | Muiruri et al. | Ama et al. | Ojiambo Wandera, Kwagala and Maniragaba |
| --- | --- | --- | --- |
| Selection Bias | M | M | S |
| Study Design | W | W | W |
| Confounders | S | S | S |
| Blinding | N/A | N/A | N/A |
| Data Collection Methods | S | S | S |
| Withdrawals and Dropouts | N/A | M | M |
| Intervention Integrity | M | M | M |
| Analysis | S | S | S |
| Global Rating | M | M | M |
